# The investigation of initial endotracheal tube cuff pressures in the operating room: a multi-center cross-sectional study in China

**DOI:** 10.1038/s41598-026-37279-3

**Published:** 2026-02-01

**Authors:** Heqi Peng, Zhaohui Tang, Yalin Li, Wei Zhang, Kaiming Duan, Hong Zheng, Lulong Bo, Yilin Zheng, Peng Wu, Jiaxin Tian, Yajuan Han, Xiaohua Zou, Gang Chen, Jun Xu, Jianqiang Guan, Xuezheng Zhang, Jianliang Sun, Yuekun Shen, Mao Zhou, Shu Zheng, Hui Wang, Hongmei Ma, Qulian Guo, Wangyuan Zou, Yingqi Weng

**Affiliations:** 1 Department of Anesthesiology, Xiangya Hospital, Central South University, 87 Xiangya Road, Changsha, China; 2 National Clinical Research center for Geriatric Disorders, Xiangya Hospital, Central South University, Changsha, China; 3 Department of Anesthesiology, People’s Hospital of Ningxiang City, Ningxiang, China; 4 Operation Office, Xiangya Public Health, Central South University, Changsha, China; 5 Department of Anesthesiology, The Third Xiangya Hospital, Central South University, Changsha, China; 6 Department of Anesthesiology, Second Xiangya Hospital, Central South University, Changsha, China; 7 Faculty of Anesthesiology, Changhai Hospital, Shanghai, China; 8 Department of Anesthesiology, Peking University First Hospital, Beijing, China; 9 Department of Anesthesiology, Daping Hospital, Army Medical University, Chongqing, China; 10 Department of Anesthesiology, The Second Affiliated Hospital of Dalian Medical University, Dalian, China; 11 Department of Anesthesiology, The Fourth Hospital of Hebei Medical University, Shijiazhuang, China; 12 Department of Anesthesiology, The Affiliated Hospital of Guizhou Medical University, Guiyang, China; 13 Department of Anesthesiology, Sir Run Run Shaw Hospital, School of Medicine, Zhejiang University, Hangzhou, China; 14 Department of Anesthesiology and Perioperative Medicine, Henan Provincial People’s Hospital, People’s Hospital of Zhengzhou University, Zhengzhou, China; 15 Department of Anesthesiology, The Third Affiliated Hospital of Sun Yat-sen University, Guangzhou, China; 16 Department of Anesthesiology, The First Affiliated Hospital of Wenzhou Medical University, Wenzhou, China; 17 Department of Anaesthesia, The Affiliated Hangzhou First People’s Hospital, Zhejiang University School of Medicine, Hangzhou, China; 18 Department of Anesthesiology, The First Affiliated Hospital of Sun Yat-sen University, Guangzhou, China; 19 Department of Anesthesiology, Sun Yat-sen Memorial Hospital, Sun Yat-sen University, Guangzhou, China; 20 Department of Anesthesiology, Fujian Provincial Hospital, Shengli Clinic Medical College of Fujian Medical University, Fuzhou, China; 21 Department of Anesthesiology, Tianjin Hospital, Tianjin, China; 22 Department of Anesthesiology, Qinghai Provincial People’s Hospital, Xining, China

**Keywords:** Endotracheal tube cuff pressure, Cross-sectional study, General anesthesia, Risk factors, Epidemiology, Risk factors

## Abstract

Cuffed endotracheal tubes (ETTs) are widely used in general anesthesia, with recommended cuff pressure (CP) between 20 and 30 cmH_2_O in adults. This multi-center study investigated actual CP management practices across 19 Grade A tertiary hospitals in China, involving 2058 anesthetized patients. The median initial CP measured within 30 min post-intubation was 48 cmH_2_O (IQR 32–70), with only 19% within the recommended range (75.4% > 30 cmH_2_O; 5.6% < 20 cmH_2_O). All institutions demonstrated similarly suboptimal CP management. Four distinct methods were used for cuff pressure estimation: pilot balloon palpation (96.2%), fixed-volume inflation (2.8%), minimal occlusive volume technique (0.7%), and direct manometry (0.2%). Among them, only direct manometry demonstrated reliable accuracy in achieving appropriate pressures. The remaining three methods showed no clear superiority over one another in terms of pressure assessment accuracy. In orotracheally intubated patients with palpation-estimated CP, independent risk factors for elevated pressure (> 30 cmH_2_O) included: age < 60 years, pressure-controlled ventilation, absence of PEEP, ETT internal diameter < 7.0 mm, aminosteroid neuromuscular blocker use, and trainee-performed inflation. These findings highlight widespread challenges in optimal CP management across Chinese tertiary care centers.

**Supplementary Information:**

The online version contains supplementary material available at 10.1038/s41598-026-37279-3.

## Introduction

Endotracheal intubation is routinely performed for patients undergoing general anesthesia. Following intubation, the endotracheal tube (ETT) cuff is immediately inflated to create an effective seal, preventing both air leakage and aspiration of oropharyngeal secretions or gastric contents. Inadequate cuff inflation increases the risk of both overt and silent aspiration, potentially leading to postoperative pulmonary complications^1^. Conversely, excessive cuff inflation compromises tracheal mucosal perfusion, resulting in ischemic injury^2,3^. Prolonged exposure to elevated cuff pressures may result in serious complications including mucosal ulceration, necrosis, tracheoesophageal fistula formation, and even tracheal rupture^4,5^. Even short-term exposure to excessive cuff pressures can induce postoperative respiratory sequelae, including cough, sore throat, hoarseness, and hemoptysis^6^. Therefore, it is widely recommended to maintain ETT cuff pressure between 20 and 30 cmH_2_O in adults with regular monitoring^6,7^. Despite these recommendations, clinical practice often relies on subjective estimation rather than objective measurement of cuff pressure. A few articles have documented the deviations from recommended cuff pressure ranges in operating room settings^5,8,9^. However, to date, no nationwide study has systematically evaluated the prevalence and potential clinical implications of this phenomenon. Multiple intraoperative factors may alter cuff pressure, including gas diffusion (particularly with N_2_O use), positional changes, iatrogenic pneumothorax or pneumoperitoneum, transesophageal echocardiography probe insertion, and direct surgical manipulation^10–14^. Nevertheless, the initial inflation pressure remains the primary determinant of cuff pressure maintenance throughout the surgical procedure.

This multicenter study was designed to: (1) quantify initial ETT cuff pressures in anesthetized patients across tertiary care centers in China; (2) evaluate current practice patterns for cuff inflation and pressure assessment; and (3) identify risk factors associated with non-compliant cuff pressures.

## Methods

This prospective, multicenter observational study was conducted between April 2019 and February 2021 across 19 tertiary care centers in major Chinese cities. All participating institutions were classified as grade A tertiary hospitals, representing the highest level of medical care in China. The study protocol received ethical approval from the Institutional Review Board of the leading center (Approval No. KE 2019010027) and was registered with the Chinese Clinical Trial Registry on March 21, 2019 (registration number: ChiCTR1900022038). In accordance with the approved protocol, written informed consent was obtained from all individual participants at each participating center. The study was conducted in full compliance with the ethical principles of the Declaration of Helsinki.

### Study population

This multicenter, cross-sectional study employed a stratified sampling approach to ensure geographic representation across Eastern, Central, and Western China. Nineteen Grade A tertiary hospitals were selected based on three key criteria: (1) geographic distribution, (2) volume of general anesthesia procedures, and (3) standardized anesthesia training programs.

**Fig. 1:**
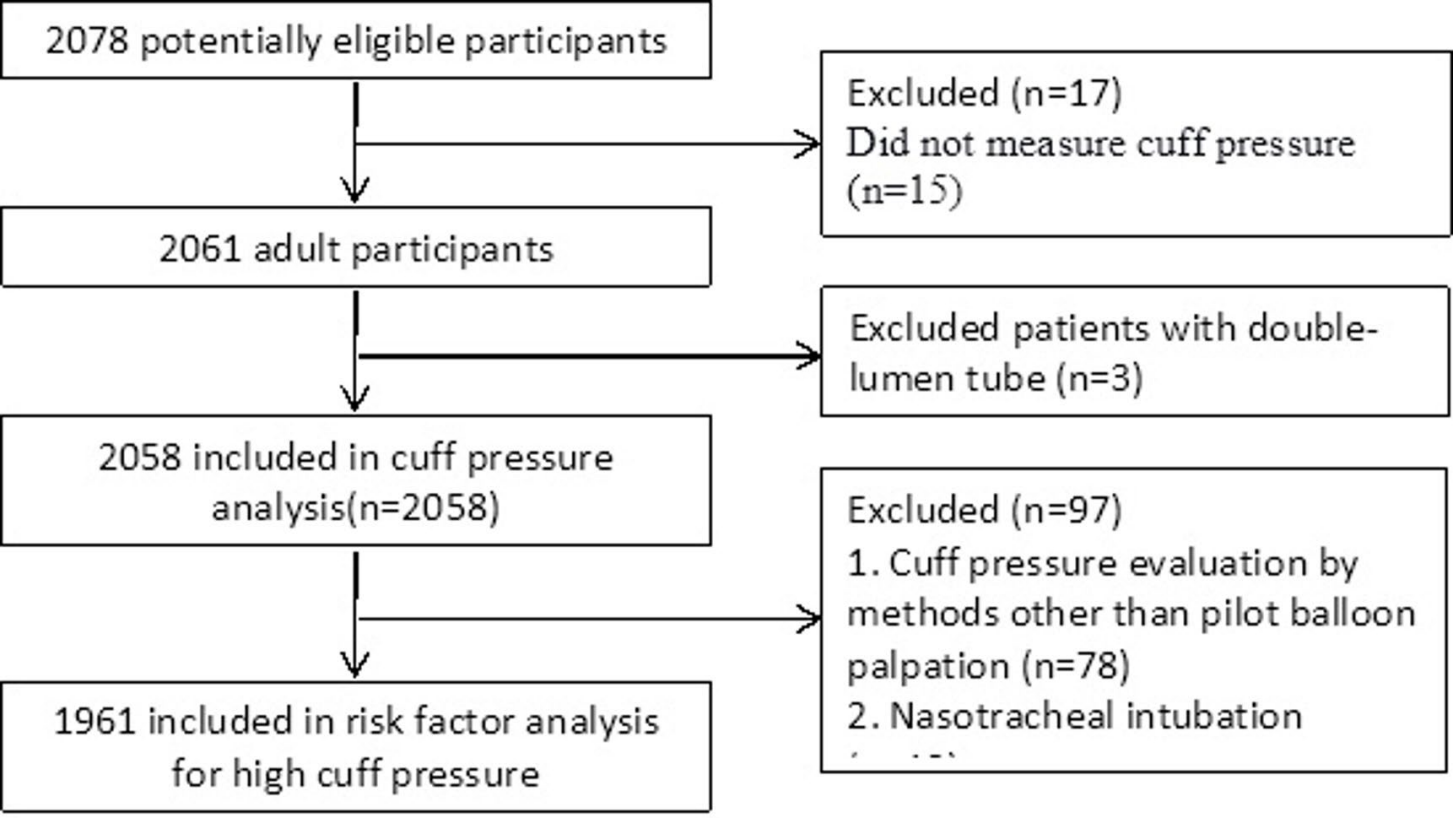
Flow-chart of participants’ enrollment.

Figure 1 illustrates the patient enrollment process. Inclusion criteria comprised: (1) age ≥ 18 years, (2) scheduled for elective surgery under general anesthesia with endotracheal intubation. (3) provision of written informed consent prior to anesthesia administration. Exclusion criteria included: (1) inability to obtain cuff pressure (CP) measurements within 30 min post-intubation; (2) nitrous oxide was used before measurement; (3) non-supine positioning, significant head/neck position changes, or surgical creation of pneumothorax/pneumoperitoneum; or (4) clinical instability precluding safe CP measurement (e.g., hemodynamic or respiratory compromise requiring immediate intervention).

Our pilot data revealed a decrease in cuff pressures for consecutive cases within the same operating room. This Hawthorne effect likely resulted from unconscious behavioral modification by anesthesia providers following initial pressure measurements. To maintain ecological validity, we implemented rigorous controls: (1) single-case enrollment per operating room per study day, (2) mandatory 5-day washout periods between study sessions, (3) complete blinding of clinical teams to measurement results, and (4) non-disclosure of study protocols until data collection concluded. These measures preserved natural clinical practice patterns while minimizing measurement bias.

### Cuff pressure measurement and adjustment

The endotracheal tube (ETT) position was first verified by the operating room anesthesiologists to ensure proper placement depth. Cuff pressure (CP) was measured using a calibrated manometer (Manometer Sensitive, VBM Medizintechnik GmbH, Germany; range: 0−120 cmH_2_O) connected to the ETT’s pilot balloon. The manometer was directly attached to the pilot balloon valve, and baseline pressure was recorded after stabilization for at least 3 s. Pressure adjustments were made by inflating or deflating the cuff through the manometer system.

A small air leak consistently occurred during manometer disconnection. To account for this expected pressure loss, the target CP was set at 30 cmH₂O during measurement to ensure the final pressure remained within the recommended 20–30 cmH₂O range after disconnection. If air leakage was detected either by audible gas escape or discrepancy between set and delivered tidal volumes, the cuff was re-inflated through the manometer under the continuous manometric monitoring until the leak was just sealed. The pressure was then briefly increased by approximately 3 cmH₂O to compensate for the small pressure drop upon disconnecting the manometer, ensuring the final in vivo pressure remained within or near the recommended range.

### Outcomes

The primary outcome was endotracheal tube cuff pressure (CP). Secondary outcomes included CP distribution and factors associated with CP > 30 cmH_2_O. Data were collected from three sources: patient records (age, sex, height, weight, surgical site), anesthesia parameters (P_peak_, PEEP, ventilation mode, anesthetics, ETT details), and inflating operator characteristics (age, sex, experience). All data were recorded on case report forms before entry into a structured Excel database for analysis. Variable selection was based on previous publications and clinical relevance.

### Sample size estimation

The sample size calculation was based on a previously reported standard deviation of 31.8 cmH_2_O for endotracheal tube cuff pressures (CP), derived from prior research^5^. With an alpha level set at 0.05 and a margin of error of 1.17 cmH_2_O (representing 5% of the standard deviation), the initial sample size requirement was determined. To account for potential 20% data loss or exclusions, the final target sample size was established at 1,844 participants.

### Statistical analysis

Missing data handling is detailed in Table S1. CPs were classified as: low (< 20 cmH_2_O), appropriate (20–30 cmH_2_O), or high (> 30 cmH_2_O). For factor analysis, participants were stratified into high CP (> 30 cmH_2_O) and non-high CP (≤ 30 cmH_2_O) groups, as high CP was the most frequently observed deviation while low CP cases were relatively rare.

Continuous variables were assessed for normality using the Kolmogorov-Smirnov test. Normally distributed data were expressed as mean ± SD and analyzed with Student’s t-test; non-normal data were reported as median (IQR) and compared using Mann-Whitney U tests. Categorical variables were presented as frequencies and analyzed with Pearson’s χ^2^ tests.

Multivariable logistic regression incorporated variables with clinical relevance and univariate *p* < 0.05. All analysis used SPSS 21.0 (IBM Corp.), with statistical significance set at *p* < 0.05 (two-tailed).

### Conference presentation

 The work was partially presented at the 28th Annual Meeting of the Chinese Society of Anesthesiology, which occurred in Changsha from September 21st to 24th, 2023.

## Results

### The ETT cuff pressures varied widely and frequently exceeded the recommended range

**Fig. 2:**
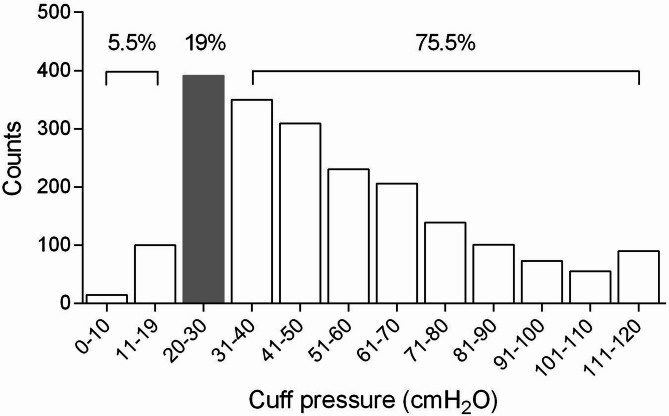
The cuff pressures distribution according to the pressure gradient.

From April 2019 to February 2021, 2058 adult participants were enrolled across 19 hospitals (939 male and 1119 female, age 53 year [42–63]; height 162.6 cm [158.0–170.0], weight 62 kg [55.00–70]; BMI 23.43 kg/m^2^ [21.22–25.71]). Body mass index was categorized for analysis according to Chinese-specific criteria: normal weight (< 24 kg/m²), overweight (24–27.9 kg/m²), and obesity (≥ 28 kg/m²)^15^. All participants were intubated with high-volume, low-pressure (HVLP) cuffed endotracheal tubes (internal diameters: 6.0–8.0 mm), which were either reinforced or non-reinforced. The measured cuff pressures showed remarkable variation, ranging from 6 to 120 cmH_2_O (with 77 measurements reaching the barometer’s upper limit recorded as 120 cmH_2_O) (Fig. 2). The median CP was 48 cmH_2_O (IQR 32–70 cmH_2_O), significantly higher than recommended levels. Strikingly, only 19% of measurements fell within the optimal 20–30 cmH_2_O range, while 75.5% exceeded 30 cmH_2_O and just 5.5% were below 20 cmH_2_O. This pattern of excessive cuff inflation was observed consistently across all participating hospitals, though with some variation between institutions (Table 1).

**Table 1:** Cuff pressures in different hospitals.

	<20 cmH_2_O *n* (%)	20–30 cmH_2_O *n* (%)	>30 cmH_2_O *n* (%)
All hospitals	114 (5.5)	391 (19.0)	1553 (75.5)
Hospital 1	4 (2.2)	19 (10.3)	162 (87.6)
Hospital 2	6 (3.3)	42 (23.2)	133 (73.5)
Hospital 3	9 (4.4)	25 (12.3)	169 (83.3)
Hospital 4	6 (6.2)	17 (17.5)	74 (76.3)
Hospital 5	2 (2.1)	6 (6.3)	87 (91.6)
Hospital 6	5 (5.2)	20 (20.6)	72 (74.2)
Hospital 7	4 (4.0)	17 (17.2)	78 (78.8)
Hospital 8	24 (23.8)	34 (33.4)	43 (42.6)
Hospital 9	10 (9.5)	21 (20.0)	74 (70.5)
Hospital 10	10 (10.6)	35 (37.2)	49 (52.1)
Hospital 11	10 (10.0)	22 (22.0)	68 (68.0)
Hospital 12	0 (0)	39 (39.0)	61 (61.0)
Hospital 13	0 (0)	9 (9.2)	89 (90.8)
Hospital 14	11 (11.0)	26 (26.0)	63 (63.0)
Hospital 15	1 (1.7)	17 (28.3)	42 (70.0)
Hospital 16	7 (7.0)	20 (20.0)	73 (73.0)
Hospital 17	0 (0)	1 (0.9)	105 (99.1)
Hospital 18	4 (3.0)	14 (14.0)	82 (82.0)
Hospital 19	1 (2.7)	7 (18.9)	29 (78.4)

## Suboptimal cuff pressure management practices in clinical settings

All illustrated in Table 2, the assessment of cuff inflation methods revealed that pilot balloon palpation was used in 96.2% of cases (1980/2058), while fixed-volume inflation (2–10 mL) was employed in 2.8% (58/2058), minimal occlusive volume technique in 0.7% (15/2058), and direct manometric measurement in only 0.2% of cases (5/2058). Among all methods evaluated, only direct pressure measurement consistently achieved proper cuff inflation pressures within the recommended 20–30 cmH₂O range. The remaining techniques failed to ensure appropriate pressure maintenance or demonstrate superior performance relative to other approaches, as evidenced by the widespread overinflation documented in our findings. This pattern highlights a critical reliance on subjective assessment methods in clinical practice, with minimal adoption of evidence-based pressure measurement techniques, underscoring the need for standardized cuff management protocols.

**Table 2:** Cuff pressures with different inflating methods.

Inflating method	Total *n* (%)	<20 cmH_2_O *n* (%)	20–30 cmH_2_O *n* (%)	>30 cmH_2_O *n* (%)
Pilot balloon palpation	1980 (96.2)	113 (99.1)	372 (95.1)	1495 (96.3)
Fixed volume	58 (2.8)	0 (0)	11 (2.8)	47 (3.0)
Minimal sealing pressure	15 (0.7)	1 (0.9)	3 (0.8)	11 (0.7)
CP measurement	5 (0.2)	0 (0)	5 (1.3)	0 (0)

### Factors associated with high ETT cuff pressure

Among the 2058 participants, 1961 cases underwent orotracheal intubation with cuff pressure estimated by pilot balloon palpation and had classifiable surgical sites, forming the primary analysis cohort. Potential risk factors for cuff pressures exceeding 30 cmH_2_O were examined in this group. Initial comparisons revealed significant differences between patients with cuff pressures ≤ 30 cmH_2_O and > 30 cmH_2_O across several variables, including patient age, ventilation mode, PEEP application, endotracheal tube internal diameter, muscle relaxant use, inflating operator experience level, and surgical site location, as detailed in Table 3.

**Table 3:** Characteristics of patients with cuff pressure ≤ 30 cmH2O and >30 cmH2O.

	Total	≤ 30 cmH2O	>30 cmH2O	*P* value
Total	1961	483	1478	
Sex, n (%)				0.919
Male	889 (45.3)	218 (45.1)	671 (45.4)	
Female	1072 (54.7)	265 (54.9)	807 (54.6)	
Age, n (%)				*0.001* ^*^
< 60 years	1305 (66.5)	291 (60.2)	1014 (68.6)	
≥ 60 years	656 (33.5)	192 (39.8)	464 (31.4)	
Over-weighted or obesity^†^, n (%)				0.691
Yes	615 (31.4)	155 (32.1)	460 (31.1)	
No	1346 (68.6)	328 (67.9)	1018 (68.9)	
Ventilation mode, n (%)				*0.003* ^*^
Volume-controlled	1862 (95.0)	471 (97.5)	1391 (94.1)	
Pressure-controlled^‡^	99 (50)	12 (2.5)	87 (5.9)	
Median peak pressure, cm H_2_O (IQR)	15 (13–17)	15 (13–17)	15 (13–17)	0.827
PEEP, n (%)				*<0.001* ^*^
With (2 cm H_2_O [2–3])	480 (24.5)	148 (30.6)	332 (22.5)	
Without	1481 (75.5)	335 (69.4)	1146 (77.5)	
Median PEEP pressure, cm H_2_O (IQR)	0 (0–0)	0 (0–0)	0 (0–2)	*0.0005* ^*^
Inhalational anesthetics, n (%)				0.550
Used	1661 (84.7)	405 (83.9)	1256 (85.0)	
Not used	300 (15.3)	78 (16.1)	222 (15.0)	
Muscle relaxant, n (%)				*0.026* ^*^
Cis atracurium or atracurium	1320 (67.3)	345 (71.4)	975 (66.0)	
Rocuronium or vecuronium	641 (32.7)	138 (28.6)	503 (34.0)	
Type of endotracheal tube, n (%)				0.228
Reinforced	1106 (56.4)	261 (54.0)	845 (57.2)	
Unreinforced	855 (43.6)	222 (46.0)	633 (42.8)	
Internal diameter, n (%)				*0.005* ^*^
< 7.0 mm	329 (16.8)	61 (12.6)	268 (18.1)	
≥ 7.0 mm	1632 (83.2)	422 (87.4)	1210 (81.9)	
Median internal diameter, mm (IQR)	7.0 (7.0–7.5.0.5)	7.0 (7.0–7.5.0.5)	7.0 (7.0–7.5.0.5)	*0.0127* ^*^
Median intubating depth, cm (IQR)	22 (22–23)	22 (22–23)	22 (22–23)	0.693
Sex of inflating operator, n (%)				0.723
Male	736 (37.5)	178 (36.9)	558 (37.8)	
Female	1225 (62.5)	305 (63.1)	920 (62.2)	
Median age of inflating operator, yr (IQR)	34 (28–43)	35 (29–42)	34 (27–43)	0.126
Professional experience, n (%)				*0.013* ^*^
Trainee^§^	722 (36.8)	155 (32.1)	567 (38.4)	
Trainer^||^	1239 (63.2)	328 (67.9)	911 (61.6)	
The surgical site, n (%)				*0.005* ^*^
Head or neck	444 (22.6)	87 (18.0)	357 (24.2)	
Other sites	1517 (77.4)	396 (82.0)	1121 (75.8)	

*P < 0.05.

† BMI ≥ 24 kg/m^2^, based on the standard in Chinese population.

^‡^ Include pressure-controlled ventilation and pressure-controlled volume-guaranteed ventilation.

^§^ Include anesthetic residents, postgraduates in anesthesiology, nurse anesthesiologists, advanced study doctors in anesthesiology, clinical interns and rotating doctors from other departments.

^||^ Include attending doctors, associate professors, and professors.

Multivariate analysis incorporated these significant factors along with additional covariates: patient sex, body mass index, peak airway pressure, endotracheal tube type (reinforced versus unreinforced), and inflating operator sex. This comprehensive adjustment identified six independent risk factors of elevated cuff pressure: patient age below 60 years, pressure-controlled ventilation mode, absence of PEEP during ventilation, administration of aminosteroid neuromuscular blocking agents, use of endotracheal tubes with internal diameter less than 7.0 mm, and cuff inflation performed by trainee anesthetists. These final adjusted associations are presented in Table 4, demonstrating the multifactorial nature of cuff overinflation in clinical practice. The findings highlight how patient characteristics, ventilation strategies, equipment selection, and inflating operator factors collectively influence cuff pressure management outcomes.

**Table 4:** Factors associated with cuff pressure exceeding 30 cmH_2_O.

	OR (95% CI)	*P* value
Sex		
Male	1.11 (0.89–1.39)	0.340
Female	Reference	
Age (classified)		
< 60 years	1.46 (1.17–1.82)	*0.001* ^*^
≥ 60 years	Reference	
Over-weighted or obesity^†^, n (%)		
Yes	0.96 (0.76–1.20)	0.704
No	Reference	
Ventilation mode		
Volume-controlled	Reference	
Pressure-controlled^‡^	2.38 (1.28–4.45)	*0.006* ^*^
PEEP		
With	Reference	
Without	1.47 (1.17–1.86)	*0.001* ^*^
Muscle relaxant		
Cis atracurium or atracurium	Reference	
Rocuronium or vecuronium	1.42 (1.12–1.79)	*0.003* ^*^
Type of endotracheal tube		
Reinforced	1.13 (0.91–1.41)	0.254
Unreinforced	Reference	
Internal diameter		
< 7.0 mm	1.58 (1.15–2.18)	*0.005* ^*^
≥ 7.0 mm	Reference	
Sex of inflating operator		
Male	1.04 (0.84–1.29)	0.^7^14
Female	Reference	
Professional experience		
Trainee^§^	1.37 (1.10–1.72)	*0.006* ^*^
Trainer^||^	Reference	
The surgical site		
Head or neck	1.25 (0.95–1.64)	0.108
Other sites	Reference	

*P < 0.05.

† BMI ≥ 24 kg/m^2^, based on the standard in China.

^‡^ Include pressure-controlled ventilation and pressure-controlled volume-guaranteed ventilation.

^§^ Include anesthetic residents, postgraduates in anesthesiology, nurse anesthesiologists, advanced study doctors in anesthesiology, clinical interns and rotating doctors from other departments.

^||^ Include attending doctors, associate professors, and professors.

## Discussion

The cuffed ETT was first described by Eisenmenger in 1893 and has since evolve to meet the needs of surgery, anesthesia, and, more recently, critical care medicine^16^. The primary function of the cuff is to form a seal against the tracheal or bronchial walls, enabling positive pressure ventilation, delivery of inhalational drugs without atmospheric pollution, and prevention of secretions, fluids, and blood from the mouth or stomach from leaking into the trachea and lungs.

When the high-pressure low-volume (HPLV) cuffed ETT was widely used, excessive CP was already recognized as a significant issue. The introduction of high-volume, low-pressure (HVLP) cuffed ETTs in the 1970 s marked a significant advancement, and these have since become the standard in clinical practice^16^. However, the low pressure in the pilot balloon can lead to over-inflation^17^. In 1984, R D Seegobin directly observed mucosal blood flow in intubated patients using fiberoptic bronchoscopes and reported that the mucosal blood flow was impaired at CPs above 30 cmH_2_O and completely obstructed at 50 cm H_2_O or higher^3^. This finding established 30 cmH_2_O as the widely accepted upper limit for appropriate CP. However, a recent study had reported airway mucosal damage in patients undergoing long-term ventilation even when CPs were maintained at 30 cmH_2_O, suggesting that a lower safety threshold for CP may be necessary for such patients.

In contrast, the lower limit of CP for HVLP ETTs is much more controversial. Park et al. suggested that a CP below 20 cmH_2_O was sufficient for tracheal occlusion based on auscultation or spirometry^18^. However, recent studies have challenged this view, indicating that a significant number of patients require CPs outside the recommended range (< 20 or > 30 mmH_2_O) to have adequate airway sealing, the required CP is influenced by the difference between the ETT cuff area and airway area^18^. Other studies have focused on the prevention of microaspiration. Rello et al. identified a CP < 20 cmH_2_O during the first eight days of intubation as an independent risk factor for ventilator-associated pneumonia (VAP)^19^. Additionally, research has shown that conventional HVLP cuffs may not prevent microaspiration even at CPs up to 60 cmH_2_O^20^. Seegobin’ s direct observations revealed that cuff folds, which may allow leakage of oral or gastric contents, persist even at CPs as high as 100 cm H_2_O^3^.

Recent advancements in ETT design have also introduced variability in the lowest CP required to prevent microaspiration, depending on cuff shape, material, and thickness^20,21^. Despite these controversies, many institutions use 20 cmH_2_O as the lower limit for acceptable CP, with the range of 20–30 cmH_2_O widely recommended for adult patients. This range balances the prevention of mucosal injury and aspiration risk, though further research is needed to optimize cuff pressure management in clinical practice^7^.

Liu et al. previously measured the CP of 236 patients immediately after intubation in four tertiary care university hospitals in Shanghai in 2010^5^, reporting a mean ETT CP of 43 ± 23.3 mmHg following estimation by pilot balloon palpation. A decade later, we conducted this study across 19 large, urban, comprehensive Grade A tertiary hospitals in China, where general anesthesia with intubation is routinely performed. Despite the anesthetic staff being well-trained and regularly updated on best practices, the findings were concerning: only a small proportion of CP measurements fell within the recommended range. Over-inflation was both prevalent and severe, with proper inflation rates varying widely among hospitals, ranging from as low as 0.9% to as high as 39%.

In our study, cuff inflation was performed using one of the following methods: pilot balloon palpation (96.2% of cases), fixed volume inflation (2–10 mL) (2.8%), minimal occlusive volume (MOV) (0.7%), Although measurement using instrumentation (e.g., a manometer) has been recommended for decades^22^, its routine use has been limited by additional costs, increased workload, and concerns regarding calibration and sanitization. Despite accumulating evidence, including our findings, highlighting its unreliability, pilot balloon palpation remains the most widely used method for cuff inflation in daily anesthetic practice worldwide. Some studies have suggested that minimal occlusive volume (MOV) is the most appropriate technique to prevent both over- and under-inflation. However, our investigation did not demonstrate its superiority over pilot balloon palpation or fixed volume inflation. Additionally, a study reported that over-inflation is common with MOV due to the difficulty in determining when to stop inflating the cuff, often resulting in excessive pressure^23^.

We also identified several factors associated with Cuff Pressure Exceeding 30 cmH_2_O. Both in vitro and in vivo studies in pediatric patients have shown that higher CPs are associated with smaller tube sizes, likely due to the inability to achieve an effective tracheal seal with a small air volume in the cuff, necessitating additional inflation^24^. Recent research in adult patients has further indicated that smaller tube sizes (e.g., ID 6.0 vs. 8.0) may increase the risk of inaccurate CP estimation by pilot balloon palpation^25^.These findings may explain why participants using endotracheal tubes (ETTs) with smaller internal diameters in our study were more likely to exhibit excessive CP. Other factors, such as differences in cuff volume between ETT sizes, were not examined in this study and cannot be ruled out as potential contributors to the observed results.

We also found that elderly patients had lower CPs, a finding consistent with a prior study in critically ill adults. The researchers attributed this to glottic muscle atrophy with age. Studies on tracheal changes in adults have demonstrated that aging is associated with increases in the tracheal anterior-posterior diameter, area, roundness distortion, and calcification scores^26,27^. These factors may change the shape of cuff in trachea or reduce the palpable softness during inflation, thus contributing to the lower cuff pressures in elderly participants comprehensively.

Prior studies indicated no correlation between inflating operator and cuff pressures^8,28^. This study, with a much larger sample size, revealed a correlation of professional experience of the inflating operator with the cuff pressures. Compared to trainees—including anesthetic residents, clinical interns, rotating doctors from other departments, nurse anesthetists, and doctors in advanced study—cuffs inflated by trainers, such as attending doctors, associate professors, and professors, were less prone to over-inflation. This better performance of trainers might be attributed to the training, clinical experience, and ongoing knowledge update^8,29,30^.

The effect of muscle relaxants on ETT CP are complex. Compared to the adductor pollicis, commonly used for neuromuscular blockade monitoring, neuromuscular blockade at the laryngeal muscles exhibits a faster onset, less intense, and more rapid recovery. Paralysis of the laryngeal muscle reduces the resting pressure of cuffs placed between the vocal cords^31^. It also diminishes or weakens swallowing and coughing, which induce CP fluctuation. In addition to blocking N2-acetylcholine receptors in skeletal muscles, muscle relaxants also bind to M2 and M3 muscarinic receptors in the airway. Their overall effect on airway constriction depends on the relative influence of antagonism of the prejunctional neural M2 muscarinic receptors, the postjunctional muscle M3 receptors, or allosteric potentiation of M3 receptors (e.g., rapacuronium)^32–34^. This could explain the discrepancies between the receptor affinities of various muscle relaxants tested in cells expressing either M2 or M3 receptors individually, and their effects on airway muscle tone in animal models and clinical settings^32–36^. Finally, histamine release by some muscle relaxants could also induce tracheobronchial spasms. The activity of smooth muscles in the proximally innervated airway may be reflected in ETT CP measurements^37,38^. Although this study observed that patients administered amino steroids muscle relaxants tended to have higher CPs than those given benzylisoquinolinium muscle relaxants, the lack of recorded dosages and precise time intervals between intubation and CP measurement complicates providing a clear explanation.

It is noteworthy that participants ventilated in Pressure-Controlled Ventilation (PCV) mode or without Positive End-Expiratory Pressure (PEEP) tended to exhibit higher CPs. Typically, both PCV mode and ventilation without PEEP are associated with lower peak airway pressures (P_peak_). An elevation in P_peak_, usually observed during laparoscopic surgeries, may induce increased CPs^11,39^. However, this was not the situation in our study, as the differences in P_peak_ were minimal (14.0 cmH_2_O in PCV vs. 15.2 cmH_2_O in VCV mode; 15.5 cmH_2_O with PEEP vs. 15.0 cmH_2_O without PEEP), and no correlation between P_peak_ and high CP was found (data not shown). Consequently, further studies were warranted to interpret these findings. Notably, for most ETTs, the incidence of micro-aspiration is inversely related to the PEEP level^40,41^. Therefore, ventilating with PEEP might be beneficial to reduce the risk of micro-aspiration and excessive CP.

Taken together, our findings highlight several key contributions. First, this multi-center study objectively quantifies a significant and persistent gap between evidence-based recommendation and clinical reality. While manometry remains the unequivocal gold standard for cuff pressure measurement, our findings reveal that manual palpation—a method shown here to be highly unreliable—is the dominant practice across diverse clinical settings. This discrepancy likely stems from non-mandatory guidelines, perceived practical barriers, and an underestimation of the impact of excessive pressure.

Crucially, this gap has demonstrable consequences. We observed a consistently high prevalence of excessive cuff pressure across all 19 participating sites, despite expected heterogeneity in patient demographics. This uniformity underscores that the issue is systemic and practice-driven. Furthermore, the risk factors we identified—such as cuff inflation by trainees and the use of specific ventilator modes—remained significant after adjusting for patient-level variables, confirming their independence from underlying case-mix variation and highlighting specific, modifiable targets for intervention.

This study has several limitations. Firstly, this study was conducted solely in high-level public hospitals in China, many of which are affiliated with medical schools or provincial medical centers. The situation in a large number of primary and secondary hospitals in China remains unknown. Secondly, we did not track postoperative complications, such as sore throat or hoarseness. This was a deliberate methodological choice: ethically, we were obliged to correct any unsafe pressure immediately, precluding a controlled comparison; scientifically, a single initial pressure is a poor proxy for the dynamic intraoperative exposure relevant to such outcomes. While establishing a direct causal link would strengthen the argument, our study provides the essential baseline data on the prevalence of the exposure (excessive pressure) that is known to cause harm. Thirdly, we did not examine the association between CPs and the material, shape, and thickness of ETT cuffs due to the wide variety of ETT types, there were 22 types of ETTs used across these institutions. Certain data were unavailable due to confidentiality agreements or because some products were discontinued.

In conclusion, ETT cuffs were frequently over-inflated in the operating room. The assessment of CP was often inaccurate, necessitating direct measurement using instrumentation. Our findings underscore that excessive cuff pressure is a multifactorial, systemic issue. They provide a compelling evidence base for implementing targeted interventions—such as mandatory manometric monitoring and structured training for trainees—to improve patient safety.

## Supplementary Information

Below is the link to the electronic supplementary material.

Supplementary Material 1

## Data Availability

The data that support the findings of this study are not openly available due to reasons of sensitivity and are available from the corresponding author upon reasonable request.
