# Supplementary material for: The investigation of initial endotracheal tube cuff pressures in the operating room: a multi-center cross-sectional study in China
Source: Sci Rep. 2026 Feb 1;16:6856. doi: 10.1038/s41598-026-37279-3 (PMC12916846; doi:10.1038/s41598-026-37279-3)
Supplement: Supplementary file 1 — Supplementary Material 1 [file 41598_2026_37279_MOESM1_ESM.docx]

Table S1. Missing data and handling (in 2058 participants)

| Variables | Missing cases | Handling method |
| --- | --- | --- |
| Height | 3 | Replaced by mean |
| Weight | 3 | Replaced by mean |
| Peak airway pressure | 2 | Replaced by mean |
| PEEP | 19 | Replaced by mode |
| Inhalational anesthetics | 3 | Replaced by mode |
| Sex of inflating operator | 1 | Replaced by mode |
| Age of inflating operator | 1 | Replaced by mean |
| Professional experience | 1 | Replaced by mode |
